# Controlled Alignment of Carbon Black Nanoparticles in Electrospun Carbon Nanofibers for Flexible Films

**DOI:** 10.3390/polym16030327

**Published:** 2024-01-25

**Authors:** Ahmed Aboalhassan, Aijaz Ahmed Babar, Nousheen Iqbal, Jianhua Yan, Mohamed El-Newehy, Jianyong Yu, Bin Ding

**Affiliations:** 1 State Key Laboratory for Modification of Chemical Fibers and Polymer Materials, College of Materials Science and Engineering, Donghua University, Shanghai 201620, China; ch_abdelkawy@hotmail.com; 2 Institute of Advanced Study, Shenzhen University, Nanhai Avenue 3688, Shenzhen 517060, China; aijaz@admin.muet.edu.pk; 3 Key Laboratory of Textile Science & Technology, College of Textile, Donghua University, Shanghai 201620, China; yanjianhua@dhu.edu.cn (J.Y.); yujy@dhu.edu.cn (J.Y.); 4 The Center of Physical Sciences, University of Science and Technology of China, Hefei 230026, China; nousheeniqbalchem@outlook.com; 5 Innovation Center for Textile Science and Technology, Donghua University, Shanghai 200051, China; 6 Department of Chemistry, College of Science, King Saud University, P.O. Box 2455, Riyadh 11451, Saudi Arabia

**Keywords:** electrospinning, carbon black surface treatment, carbon nanocomposite bendable carbon nanofiber mats, flexibility mechanism

## Abstract

Carbon nanofiber (CNF) films or mats have great conductivity and thermal stability and are widely used in different technological processes. Among all the fabrication methods, electrospinning is a simple yet effective technique for preparing CNF mats, but the electrospun CNF mats are often brittle. Here, we report a feasible protocol by which to control the alignment of carbon black nanoparticles (CB NPs) within CNF to enhance the flexibility. The CB NPs (~45 nm) are treated with non-ionic surfactant Triton-X 100 (TX) prior to being blended with a solution containing poly(vinyl butyral) and polyacrylonitrile, followed by electrospinning and then carbonization. The optimized CB-TX@CNF mat has a boosted elongation from 2.25% of pure CNF to 2.49%. On the contrary, the untreated CB loaded in CNF displayed a lower elongation of 1.85% because of the aggregated CB spots created weak joints. The controlled and uniform dispersion of CB NPs helped to scatter the applied bending force in the softness test. This feasible protocol paves the way for using these facile surface-treated CB NPs as a commercial reinforcement for producing flexible CNF films.

## 1. Introduction

Recently, the massive growing demand for flexible electronic gadgets has driven researchers to develop ultra-flexible and lightweight devices [1,2,3]. To realize such flexible devices, electrolytes, a polymeric separator, bendable electrodes, and the device case, among other items, are required. For electrodes, CNF mats are believed to be among the highly promising materials capable of producing free-standing flexible electrodes due to their interconnected structure integrity, chemical stability, and electronic conductivity [4]. Benefiting from these inherent characteristic features of CNF, many material scientists have reported the development of numerous composite CNF-mats-based electrodes for making flexible Li-ion, Na-ion, and Li-S batteries, as well as supercapacitors and other energy storage devices [5,6]. However, the flexibility of CNF mats is still insufficient from a commercial production perspective, which has stimulated scientists to develop novel protocols for fabricating reliable soft CNF [7,8].

Amongst the various reported strategies, electrospinning is the most facile method with which to produce an endless and interconnected nanofibrous network to realize trustworthy, flexible CNF electrodes [9]. Nevertheless, CNF networks produced using electrospinning often suffer from low mechanical stability, especially when subjected to an applied bending force. Indeed, regular or prolonged exposure to these forces induces cracks, propagates them, and eventually destroys the structure of the CNF network. Therefore, many research groups are racing to employ nano-reinforcements to support CNF against the applied forces and boost their mechanical stability to meet the market demand for soft electrodes [10,11]. Plenty of carbon nanostructures with various dimensions have been investigated as reinforcements for CNF, such as 1D carbon nanotubes (CNTs), 2D graphene, graphene oxide (GO), and other analogues [12,13,14]. Notably, 1D materials such as carbon nanotubes have been reported to suffer from self-aggregation, leading to irregular distribution within the CNF structure/matrix, which affects its mechanical performance [12]. For example, one of the most common strategies with which to solve the aggregation issue of CNTs is to make chemical graphitizing similar to the previously reported amine-terminated polyphosphazene functionalized on the CNTs to be subsequently reinforced in lignin-based electrospun CNF mats [15]. The functionalization protocols are costly and ineffective and usually employ harsh chemicals which are not applicable for large-scale production [14,15].

On the other hand, 2D materials, for instance, MXenes, graphene, and their analogues, do not fit well in a 1D nanofibrous structure and partially escape the fibrous matrix, thus leading to anisotropic mechanical stability [13,16]. The main limitation encountered in incorporating CNF with 2D nanomaterials is their ability to delaminate out of the CNF skeleton. Besides aggregations, the numerous disadvantages of using MXene when loaded into the CNF, such as MXene self-restacking within the polymeric solution, account for the exposed portion of MXene emerging from the CNF nanofibrous skeleton [17,18]. So, because both 1D and 2D nanomaterials show numerous issues when loaded in the CNF, using 0D nanomaterials, dispersed in the CNF, is highly recommended to overcome these issues.

From 0D nanomaterials, carbonaceous 0D nanomaterials are believed to have an edge over their counterparts when it comes to tailoring the mechanical characteristics of CNF networks. There is a diverse range of carbonaceous 0D nanomaterials, which include carbon quantum dots, carbon nano onions, fullerene, and carbon black nanoparticles (CB NPs) [19,20]. CB NPs offer many advantages, such as easy and abundant commercial availability, low cost, and a well-established production process with high precursor abundance [21]. Therefore, CB NPs have been successfully employed in a wide variety of applications, including tire production, catalyst support, and as an industrial filling agent, conductivity additive in electrode fabrications, and polymer nano-reinforcement [22]. Besides these advantages, the size of commercial CB NPs as 0D NPs makes them the best candidate for reinforcement in the CNF matrix without affecting its morphology. However, CB NPs suffer from a high tendency to form agglomerates when it comes to polymer reinforcements; hence, forming a uniform dispersion of CB NPs in polymeric solutions is crucial and challenging [22]. To do so, several protocols have been developed with which to modify the surface of CB such as via surface grafting with a hyper-branched polymer, radiation superficial treatment, high-energy electron beam irradiation or S-doping, and pore-structure modification [23,24,25]. However, the complex nature of these strategies hinders their acceptability and makes them unfeasible for producing CNF reinforced with CB NPs.

In this study, we propose a feasible and scalable protocol with which to enhance the mechanical properties of CNF by ensuring that the CB NPs dispersion is well-aligned within the nanofibrous structure. To achieve this goal, CB NPs were first pretreated with a non-ionic surfactant Triton-X 100 (TX) to modulate their surfaces. Afterwards, the treated CB NPs were used to generate a uniform suspension of CB NPs in a blended polyvinyl butyral and polyacrylonitrile (PVB/PAN) polymeric solution. Consequently, electrospinning of the homogenous solution of CB@PVB-PAN produced a controlled orientation of CB NPs in the resultant fibers, which further led to the superior mechanical performance of these fibers compared to pure CNFs and those produced with untreated CB NPs. This study opens the possibility of using this generally feasible protocol to fabricate ultra-flexible and bendable CNFs for practical usage as soft electrodes.

## 2. Materials and Methods

### 2.1. Materials

Polyacrylonitrile (PAN, *M_w_* = 90 kDa) and polyethylene glycol tert-octylphenyl ether (Triton-X 100 or TX-100) were purchased from Sigma-Aldrich (Shanghai, China). *N*, *N*-Dimethyl formamide (DMF) was obtained from the Shanghai Chemical Reagents Co., Ltd., Shanghai, China. Poly(vinyl butyral) copolymer (PVB, *M_w_* = 170~250 kDa) and carbon black nanoparticles (with an average diameter of ~45 nm, acetylene black type) were obtained from Aladdin Chemistry Co., Ltd., Shanghai, China. All chemicals were of analytical grade and used as received without further purification.

### 2.2. Surface Pre-Treatment and Electrospinning Precursor Solution

For the surface treatment of CB NPs, 0.27 mM of TX-100 was first dissolved in DMF. Afterwards, 2 wt.% of CB NPs were dispersed in TX-DMF solution via normal bath sonication for 30 min until a visibly uniform suspension of CB-TX in DMF was achieved. Subsequently, PVB (2 wt.%) powder was slowly poured into the CB-TX suspension and stirred for 30 min. Later on, PAN (8 wt.%) powder was gradually added to the CB-TX-PVB suspension and stirred vigorously for another 8 h at room temperature to ensure that PAN was completely dissolved. To emphasize the uniform dispersion of CB NPs within the composite polymeric solution, the solution was subjected to bath-sonication for another 30 min prior to the electrospinning process. For the non-treated CB NPs, the same procedure was applied in the absence of TX-100. Moreover, a control sample of the pure polymeric blend was also prepared for comparative analysis. The polymeric blending ratio of PVB to PAN solution in DMF was maintained at a fixed ratio of 2 to 8 wt.% relative to the solution’s total weight.

### 2.3. Electrospinning Process and Thermal Treatments

The electrospinning process was carried out by using the DXES-3 spinning machine (SOF Nanotechnology Co., Ltd., Guangzhou, China). The feed rate of solutions was set at 1.5 mL/h. As a high voltage of 25 kV was applied to the needles, numerous continuous nanofibers were sequentially generated. These nanofibers were collected on aluminum foil wrapped around a grounded rotating metallic cylinder, which was placed at a distance of 20 cm from the needle tip. The temperature and humidity of the spinning chamber were controlled at 25 ± 1 °C and 50 ± 5%, respectively. The as-obtained precursor fibers were immediately dried at 60 °C for 1 h in a vacuum oven to remove the remaining solvent and then pre-calcined at 280 °C for 2 h in air. Thereafter, the stabilized polymeric nanofiber mats were annealed under high-purity N_2_ gas (99.999%) at 850 °C for 2 h with a ramping rate of 2 °C/min. Resultant fibrous samples were labeled as CNF, CB@CNF, and CB-TX@CNF, corresponding to pure CNF, untreated CB in CNF, and surface-treated CB in CNF, respectively.

### 2.4. Characterizations

The morphology analysis of the fabricated carbon nanofiber films and/or mats and the distribution of CB NPs within the CNF structures were monitored via high-resolution field emission scanning electron microscopy (FE-SEM), employing the model of S-4800 (Hitachi Ltd., Tokyo, Japan). For the carbonous microstructure investigations, X-ray powder diffraction (XRD) an (Tongda, TD-3500, Dandong City, China) with a source Cu K_α_ of λ = 1.5406 Å and a micro-Raman spectroscopy system (inVia-Reflex, Renishaw, Co., New Mills, UK) were employed for each sample. For the surface chemical composition of the fabricated samples, X-ray photoelectron spectroscopy was conducted for the three samples. The X-ray photoelectron spectroscopy (X-ray Thermo Fischer, ESCALAB 250 Xi, supplier from Shanghai, China) was employed under specific conditions. Among them, the analytical chamber vacuum was 8 × l0^−10^ Pa, and the excitation source was Al-K_α_ (hv = 1486.6 eV). The work voltage was set as 12.5 kV, and the filament current was as 16 mA. Ten cycles of signal accumulation were carried out. The sample was etched with an argon ion gun. The etched spot size was 1.5 mm, the etched voltage was 3000 eV, and the etched time was 4500 S. The pass-energy was 40 eV, the step length was 0.1 eV, and C1S = 284.80 eV was used as the energy standard for charge correction. Furthermore, the tensile mechanical properties and softness of the fabricated mats were inspected by operating both a tensile tester (XQ-1A, Fiber Tensile Tester, Shanghai New Fiber Instrument Co., Ltd., Shanghai, China) according to the international standard (ISO 1798:2008 [26]) and a bending rigidity tester (RRY-1000, Hangzhou Qingtong & Boke Automation Technology Co., Ltd., Hangzhou, China) according to the ASTM D 2923-95 [27].

## 3. Results and Discussion

**Figure 1:**
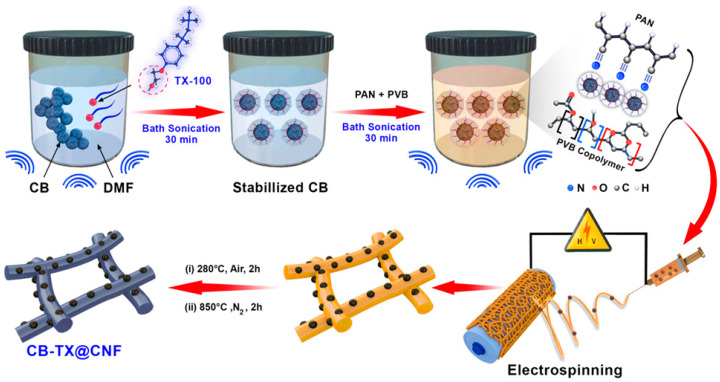
Schematic illustration for the fabrication process of flexible CB-TX@CNF films.

Figure 1 displays the schematic fabrication diagram of our proposed simple strategy, starting with the surface treatment of CB by Triton X-100, (TX), a surfactant to obtain CB-TX stabilized particles, before mixing it with the PAN/PVB polymeric solution. Although this treatment step is simple, it is important to let the use of CB NPs as a reinforcement in CNF be applicable without facing aggregation issues. Notably, it is expected that the uniform alignment of CB NPs in CNF skeleton will widely enhance the graphitization degree of the deduced mats, resulting in boosting the mechanical properties [28].

After the surface treatment of CB-TX and subsequent loading of it into the polymeric solution, the electrospinning process is begun. Afterwards, the three samples (pure CNF, CB@CNF, and the treated CB-TX@CNF) were subjected to the same pre-optimized thermal treatments of peroxidation at 280 °C in air and then carbonized at 850 °C in N_2_, whereby graphitic layers were formed within the CNF microstructure, further providing acceptable mechanical stability [11]. 

**Figure 2:**
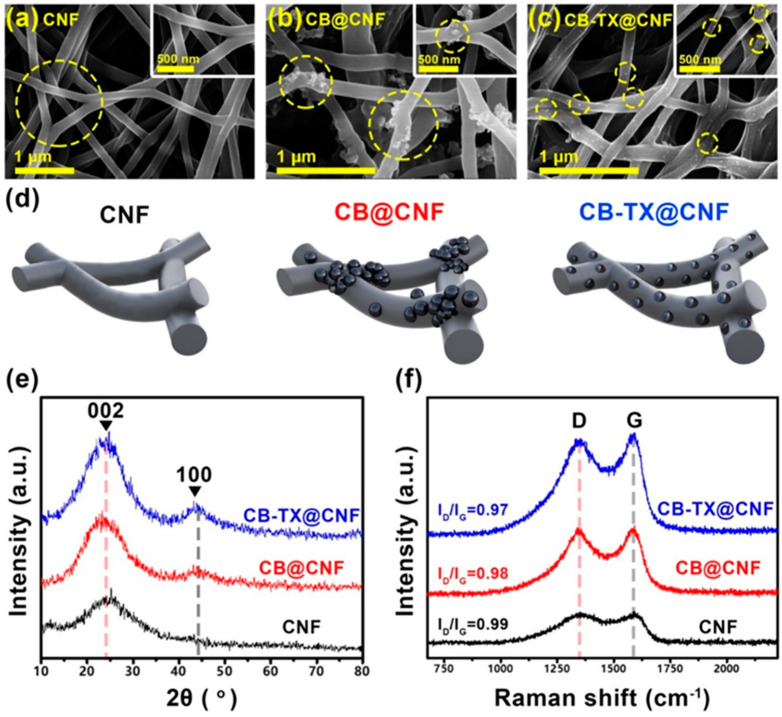
(**a**–**c**) FE-SEM of the pure CNF, CB @CNF, and CB-TX@CNF, samples, respectively. (**d**) Schematic illustration showing the particles distribution in samples of CNF, CB@CNF, and CB-TX@CNF; (**e**) XRD spectra; and (**f**) Raman spectra of CNF, CB@CNF, and CB-TX@CNF.

To prove the CB NPs distribution homogeneity in the CNF skeleton, FE-SEM was employed to explore CB positions along with CNF morphology, as shown in Figure 2a–c. The pure CNF, made of PVB-PAN, displayed an excellent interconnected and randomly oriented fibrous structure (Figure 2a). The fibers were smooth and cylindrical in shape. In contrast, the non-treated CB@CNF exhibited an interconnected fibrous structure similar to pure CNF; however, numerous agglomerates in the CNF matrix were obvious in CB@CNF (Figure 2b), which was attributed to the uncontrolled distribution of CB NPs in the polymer before the electrospinning process [29]. Interestingly, CB-TX@CNF revealed a regulated distribution in the nanofibrous structure without affecting the whole interconnected fibrous network (Figure 2c). CB NPs were uniformly aligned along the fiber axis with no apparent aggregation, and no beads were formed within the nanofibrous structure, which was credited to the uniform dispersion of CB in the composite solution. This was tanks to TX-100 pre-treatment on the NPs’ surface, which made achieving this even and uniform dispersion possible. TX-100, as a surface modulator for CB/polymer mixture, was chosen because of its nature as a non-ionic surfactant, which ensured no significant effect on the spinning polymeric solution properties, and as a result, it led to the maintenance of the nanofiber skeleton of the produced CNF [30]. To simplify the resultant samples, Figure 2d shows the schematic illustration of corresponding pure CNF, CB@CNF, and CB-TX@CNF. The CB-TX@CNF sample was similar to CB@CNF but with the addition of TX, which helped to control the homogeneity of the dispersed CB. 

XRD spectra of the samples were employed to analyze the consequence of the controlled alignment of CB NPs on CNF microstructure. Figure 2e shows two broad peaks at 24° and 43° that were analogous to the diffraction planes of (002) and (100) in graphitic carbon [31]. CB-TX@CNF displayed a higher degree of crystallinity at (100) plane than pure CNF because of the well-aligned CB NPs that acted as catalytic points to induce more graphitization all over the structure [32]. Moreover, these uniform CB catalytic points were more distributed in the CNF structure than the aggregated ones, which were in specific spots and had a lower effect than the well-distributed ones; hence, the CB-TX@CNF had more crystallinity than CB@CNF and CNF. To further explore the graphitization degrees, Raman scattering was conducted for all samples (Figure 2f). The D-band (1345 cm^−1^) and G-band (1580 cm^−1^) in all samples represented the disordered amorphous carbon and ordered graphitic *sp*^2^ hybridization carbons, respectively [33]. The disordered I_D_/I_G_ degrees of CB-TX@CNF, CB @CNF, and pure CNF were 0.97, 0.98, and 0.99, respectively. This increment in the disorder degree represents the decrease in graphitization, which means that CB-TX@CNF had the highest degree of graphitization, and this is well-matched with the above XRD results. This high graphitization in the microstructure of CB-TX@CNF is mainly attributed to the uniform distribution of CB NPs as catalytic graphitization spots all over CNF, while other samples have no such abundance of catalytic spots [34].

**Figure 3:**
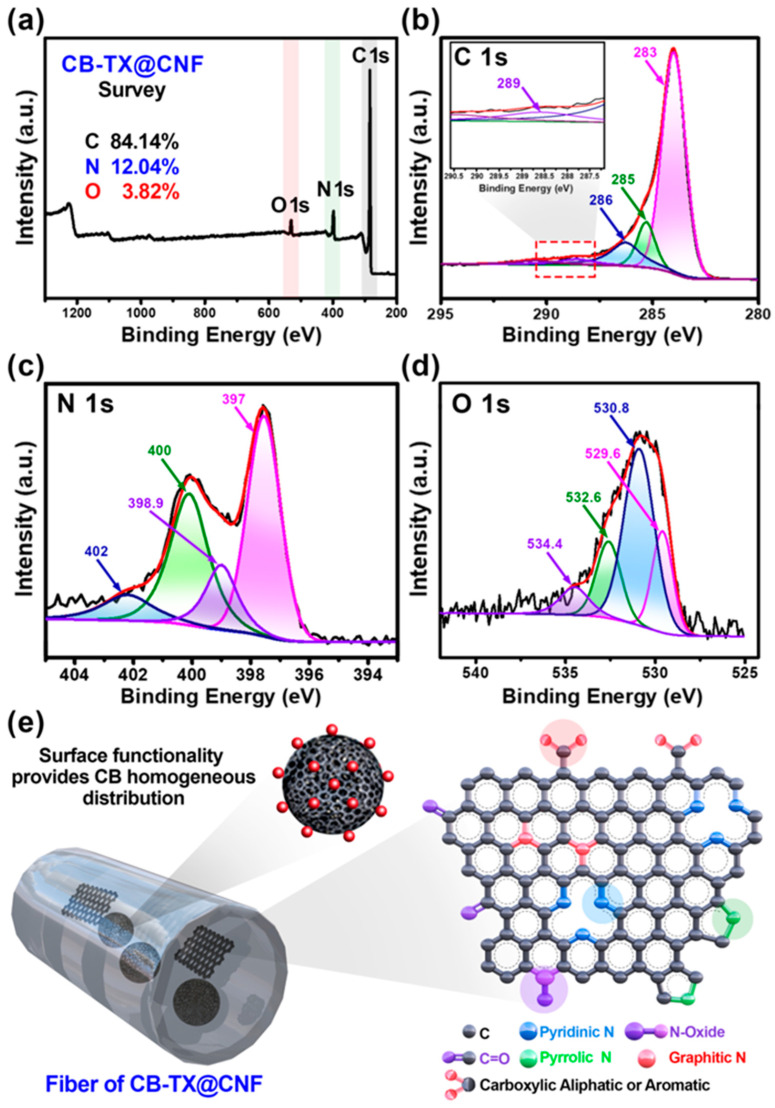
(**a**) XPS survey of the optimized sample of CB-TX@CNF and (**b**–**d**) C 1s, N 1s, and O 1s deconvolution plots. (**e**) Schematic illustration of the surface functionalities over the CB-TX, the graphitic moieties within CB-TX@CNF, and a sketch of the doping atoms within the CNF structure.

The chemical nature and the composition of O and N doping inside the optimized CB-TX@CNF sample were evaluated via X-ray photoemission spectroscopy (XPS). XPS survey scan (Figure 3a) showed the existence of three major peaks: graphitic C 1s peak at 284.6 eV, N 1s peak at 400 eV, and weak O 1s peak at 530 eV, indicating the co-doping of O and N into the skeleton of CNF. The atomic ratios of carbon, nitrogen, and oxygen were 84.14, 12.04, and 3.82%, respectively, which indicated the enrichment of CB-TX@CNF with co-doping by N and O [33]. The high-resolution XPS for C 1s in the range of 280–290 eV (Figure 3b) exhibited four peaks via the deconvolution fitting of the C 1s spectrum. The major peak at 283.4 eV was accounted for by the *sp*^2^ carbon atoms (C1)-constituted graphitic regions, which indicated that most of C atoms were located in a honeycomb lattice [35]. The other three weak peaks at 285, 286, and 289 eV corresponded to the *sp*^3^ C, C-N, and O=C-O, respectively, which confirmed the incorporation of the oxygen and nitrogen into the carbon moiety of CB-TX@CNF [36]. The N 1s peak could be deconvoluted into four individual peaks (Figure 3c) at 397 eV, 398.9, 400 eV, and 402 eV assigned to pyridinic, pyrrolic, graphitic, and N-oxide, respectively [37]. Moreover, the O 1s spectrum could be deconvoluted into four sub-peaks (Figure 3d) at 529.6, 530.8, 532.6, and 534.4 eV, which could be credited to the presence of diverse oxygen functionalities, such as C=O aromatic, C-O aliphatic, C-O aromatic, and O=C-O, respectively [36]. The N-doping source was from PAN, while the O-doping mostly comes from PVB and TX-100 [36]. Based on these analyses, Figure 3e displays a simplified schematic illustration for the proposed N and O co-doped CB-TX@CNF structure and the importance of surface functionalities on the CB and the graphitic domains within CNF, which provides good distribution of CB NPs and the associated graphitized domains [38]. N-doping as pyridinic, pyrrolic, graphitic, and N-oxide was found in high content in the sample of CB-TX@CNF (as concluded from the XPS of this membrane), implying the availability of diverse electrochemically active sites for energy storage devices such as soft batteries and supercapacitors [35,38,39].

**Figure 4:**
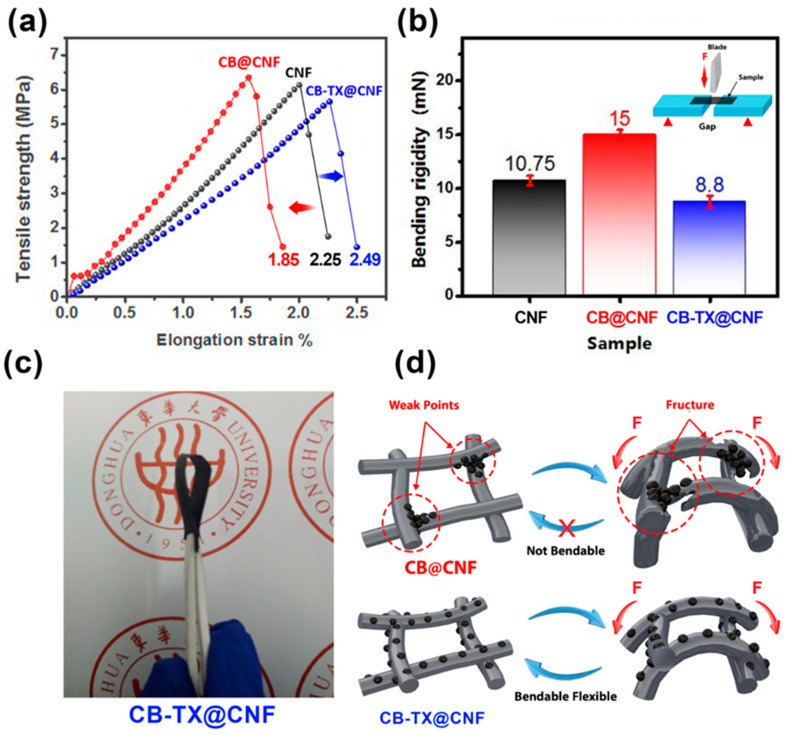
Mechanical performance of the as-fabricated samples. (**a**) The curve of the mechanical tensile strength versus the elongation strain. (**b**) The bending stiffness of CNF, CB@CNF, and CB-TX@CNF samples, respectively (the inset is the bending rigidity test representation sketch). (**c**) Photo image of the bent film of CB-TX@CNF. (**d**) Schematic illustration of the mechanism of applied force absorption in both samples of the treated CB-TX@CNF and the non-treated CB@CNF.

The mechanical properties of the fabricated CNF films are shown in Figure 4. It is apparent from Figure 4a that the pure CNF (PAN/PVB) displayed a decent elongation of 2.25%; however, it drastically dropped to 1.85% for CB@CNF (the sample prepared with untreated CB). This severe drop in elongation strain is ascribed to the agglomeration of untreated CB NPs at different sites creating weak spots in the nanofibrous matrix. Remarkably, the elongation for CB-TX@CNF significantly increased to 2.49%, which could be credited to the uniform distribution of CB-TX within the CNF axis. Additionally, lots of inter-joints bonding in CB-TX@CNF provided strength to the nanofibrous network and hence improved the elongation strain [38]. Figure 4b displays the ability of CNF mats to bear applied bending force via a three-point bending rigidity test (the inset sketch of Figure 4b). Pure CNF requires a certain bending force of ~10.7 mN to bend the film. Interestingly, CB-TX@CNF film required less force of ~8.8 mN, which means that this film was softer than CNF and could bear more bending forces without breakage [38]. Of note, the uniform CB distribution in CB-TX@CNF nanofiber skeleton could evenly scatter the applied force, thus enhancing flexibility and softness. Notably, in the case of the untreated CB, the CB@CNF sample required bending force with an increase in the value to 15 mN, which was more than the pure CNF. The increment in the required force means that CB@CNF was the most rigid sample due to the aggregated CB spots within the fiber matrix. Therefore, the enhancement of the softness behavior with TX-100 content was limited to CB-TX@CNF. Figure 4c is the digital image of CB-TX@CNF film and demonstrates its ability to easily withstand the bending force when bent to 180°. This overall improved mechanical performance of CB-TX@CNF film could be ascribed to its highly interconnected fibrous structure and the controlled, well-distributed CB NPs in the fiber matrix, which helped maintain their integrity and mechanical stability even under applied forces and various bending statuses, which could be beneficial for soft electronic applications [38,39].

To understand the flexibility mechanism through the correlations of the morphological–mechanical behavior of the sample CB-TX@CNF in parallel to the non-treated CB@CNF, we proposed Figure 4d, which schematically demonstrates the flexibility mechanism comparison. When an external bending force (F) is applied on CB@CNF, it shows low bending flexibility due to the presence of aggregated CB NPs within the fibrous structure, which further creates weak spots at which CNF can easily become cracked and damaged, as hypothesized in the upper part of Figure 4d. On the other hand, when the force is applied on CB-TX@CNF, the even alignment of CB over CNF provides uniform force absorption. Moreover, the graphitic domains formed due to the uniform CB distribution can act like a mud-brick structure; therefore, the corresponding film displays more softness and bendability than the other samples. This boosted flexibility makes CB-TX@CNF film one of the premium candidates for application in soft portable energy storage devices [40].

## 4. Conclusions

In this study, we have developed a feasible protocol by which to enhance the mechanical properties of CNF mats by controlling the alignment of CB NPs. To this end, CB NPs were first treated with the non-ionic surfactant Triton-X 100 (TX), and later, a composite solution comprising CB-TX-stabilized NPs and PVB-PAN was developed by rigorous stirring and bath-sonication. Electrospinning this homogenous composite solution with carefully designed process parameters, the uniform dispersion of CB NPs in the fiber matrix of the resultant beads-free nanofibrous film was achieved. Optimized CB-TX@CNF film displayed superior mechanical elasticity and bendability to the pure CNF and CB@CNF (i.e., CNF film with untreated CB) samples. The elongation of the CB-TX@CNF film was significantly boosted to 2.49% compared to 2.25% for pure CNF and 1.85% for untreated CB@CNF. This improved elongation was credited to the controlled dispersion of CB NPs, which helped to scatter the applied bending force during the mechanical stress–strain and softness tests. This protocol can be employed for loading various NPs in CNF films and opens new possibilities for developing flexible CNF films with enhanced mechanical properties.

## Data Availability

The data presented in this study are available on request from the corresponding author.
